# Does Transperineal Prostate Biopsy Affect Sexual Function? Results from a Prospective Cohort Study

**DOI:** 10.3390/jcm15031260

**Published:** 2026-02-05

**Authors:** Kursat Kucuker, Alper Simsek, Mehmet Kirdar, Burak Saglam, Oguz Celik, Murat Can Erdogan, Ilker Gokcedag, Mesut Berkan Duran, Aykut Akinci, Caner Ozdemir, Yusuf Ozlulerden, Sinan Celen

**Affiliations:** 1 Department of Urology, School of Medicine, Pamukkale University, Kınıklı, Denizli 20070, Türkiye; 2 Department of Urology, School of Medicine, Bandırma Onyedi Eylül University, Balikesir 10250, Türkiye; 3 Department of Urology, Bandırma Training and Research Hospital, Balikesir 10200, Türkiye; 4 Republic of Türkiye-Ministry of Health, Ankara 06800, Türkiye

**Keywords:** prostate cancer, prostate needle biopsy, transperineal prostate biopsy, erectile dysfunction, ejaculatory dysfunction

## Abstract

**Objectives:** Transperineal (TP) prostate biopsy is increasingly used because of its lower complication rates compared with the transrectal approach. However, prospective data regarding its effects on erectile and ejaculatory function remain limited. This study prospectively evaluated short-term sexual function outcomes after TP prostate biopsy in sexually active men. **Methods:** This single-center prospective observational cohort study included men undergoing TP prostate biopsy between 15 April 2025 and 1 September 2025. Indications for biopsy were prostate-specific antigen levels >4 ng/mL, abnormal digital rectal examination findings, or suspicious lesions (PI-RADS ≥ 3) on multiparametric prostate MRI. Sexual function was assessed at baseline and at 1 and 3 months after biopsy using the International Index of Erectile Function (IIEF-5), the Premature Ejaculation Diagnostic Tool (PEDT), and the Male Sexual Health Questionnaire–Ejaculatory Dysfunction Short Form (MSHQ-EjD-SF). **Results:** Overall, 249 sexually active men were analyzed. No significant changes in erectile or ejaculatory function were observed in the overall cohort at either follow-up point. In contrast, among 132 men diagnosed with prostate cancer, significant declines were observed in IIEF-5, PEDT, and MSHQ-EjD-SF scores at both 1 and 3 months compared to baseline (all *p* < 0.001). **Conclusions:** Transperineal prostate biopsy minimally affects sexual function in the general population. However, prostate cancer patients experience notable deterioration in erectile and ejaculatory outcomes, which may be a transient decline, and long-term follow-up is necessary for this subgroup.

## 1. Introduction

Worldwide cancer surveillance data indicate that more than one million men are newly diagnosed with prostate cancer (PCa) each year, with annual mortality exceeding 350,000, placing it among the most common malignancies in men [1]. The incidence of PCa is expected to increase with increasing age, and the frequency of prostate biopsies is also expected to increase. Before starting treatment, a biopsy is necessary to confirm the diagnosis of prostate cancer histologically. Although prostate cancer is prevalent worldwide, the detection method and diagnostic technology remain controversial. Prostate biopsy for cancer diagnosis can be performed using either the transperineal (TP) or transrectal (TR) approach. The transrectal approach to prostate biopsy carries a risk of infectious and hemorrhagic complications, including rectal bleeding, fever, sepsis, hematuria, and acute urinary retention, and has been associated with significant false-negative rates, particularly for anterior and apical tumors [2,3,4]. Transperineal prostate biopsy (TP-Bx) has increasingly been adopted as an alternative to the transrectal approach due to its lower rate of infectious and hemorrhagic complications. The PREVENT study, which included 658 patients, compared the effect of biopsy route on infectious complications. Infectious complications were found to be significantly higher in transrectal biopsy compared to transperineal biopsy [5].

Erectile dysfunction (ED) has also been reported after prostate biopsy [6]. Several factors have been proposed to influence post-biopsy erectile outcomes, including patient age, neurovascular bundle injury during local anesthetic injection, compression from hematoma or edema, the number of biopsy cores taken, previous biopsy sessions, procedure-related anxiety, biopsy type (transperineal or transrectal), and the time interval after the procedure [7,8,9,10,11]. Anatomically, laterally directed biopsy needles may cause neurovascular trauma, leading to transient neurapraxia, local edema, or hematoma formation within the neurovascular bed of the prostate. Such microvascular and neural injuries could result in temporary impairment of erectile function. Furthermore, considering that transperineal biopsies often involve multiple needle passages through the prostatic apex, where the neurovascular bundles converge, this approach may theoretically carry a higher risk of neurapraxia-related erectile dysfunction compared with the transrectal route. Nevertheless, prospective data on the sexual side effects of transperineal biopsy remain limited, particularly regarding the separation of procedure-related effects from diagnosis-related psychological stress [3].

Transient ED or ejaculatory dysfunction (EjD) are possible complications after prostate biopsies. Studies on ED and EjD after prostate biopsy are full of conflicting results. In this prospective study, we investigated whether transperineal prostate biopsy is associated with erectile and/or ejaculatory dysfunction in sexually active men using validated questionnaires.

## 2. Materials and Methods

This prospective observational cohort study was employed at the Department of Urology, Pamukkale University Faculty of Medicine, between 15 April 2025 and 1 September 2025. The design, conduct, and reporting of this observational study followed the principles outlined in the STROBE statement. Ethical approval was ensured according to the Declaration of Helsinki. Patients were informed in detail about the study design, objectives, and potential risks, and all provided written informed consent before enrollment. The conduct of this research was authorized by the Pamukkale University Clinical Research Ethics Committee (Approval No: E-60116787-020-677999). This was a prospective observational cohort study and did not involve any experimental intervention; therefore, clinical trial registration was not required.

No generative artificial intelligence tools were used for data analysis or interpretation. AI-assisted tools were used solely for language editing to improve grammar, clarity, and stylistic consistency.

Indications for biopsy included serum prostate-specific antigen (PSA) levels > 4 ng/mL, abnormal digital rectal examination findings (e.g., hard nodules), or multiparametric prostate MRI (Magnetic Resonance Imaging) findings classified as PI-RADS ≥ 3.

Exclusion criteria included uremia, uncontrolled hypertension, bleeding diathesis, use of unadjusted anticoagulant or antiplatelet therapy, or active urinary tract infection. Patients who underwent any active prostate cancer treatment (radical prostatectomy, radiotherapy, or androgen-deprivation therapy) within the three-month follow-up period were also excluded. In our institution, histopathological evaluation of prostate biopsy specimens typically takes 4–6 weeks, and PSMA PET imaging for patients with malignant pathology is usually completed within an additional 6–8 weeks. Consequently, none of the patients had initiated active oncological treatment during the three-month follow-up period. Additionally, PDE5 inhibitor use was assessed through both direct patient inquiry at baseline and at each follow-up visit, and verification of medical records including prescription data. Patients using PDE5 inhibitors were excluded from the study. Patients were considered ‘sexually active’ if they reported having a regular sexual partner, engaged in sexual intercourse at least once per week in last 4 weeks, and were able to achieve orgasm and ejaculation during sexual activity. These criteria ensured the validity of ejaculatory function assessments, as the PEDT and MSHQ-EjD questionnaires require recent ejaculatory experience for accurate scoring.

**Figure 1:**
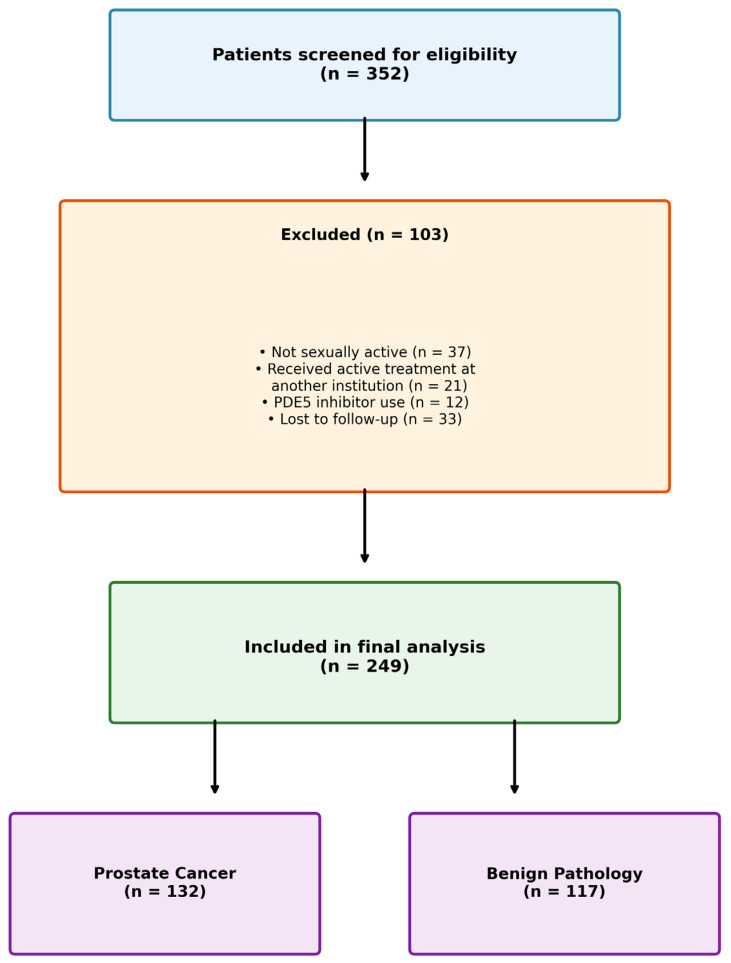
Patient flow diagram showing screening, exclusion, and final study population.

A total of 352 patients who underwent TP prostate biopsy were screened for eligibility. Of these, 103 patients were excluded: 37 were not sexually active, 21 received active treatment for PCa at another institution, 12 were using PDE5 inhibitors, and 33 were lost to follow-up. The remaining 249 patients were included in the final analysis (Figure 1).

Demographic and clinical data including age, PSA level, number of biopsy cores, and pathological outcomes were recorded. Sexual function was assessed using the Turkish validated version of the IIEF-5, the Turkish version of the PEDT, and the MSHQ-EjD at baseline, and at 1 and 3 months after biopsy [12,13,14]. Erectile function was evaluated using the total IIEF-5 score numeric variable. Patients were not grouped according to categorical severity levels, as the primary aim was to assess score changes over time.

Baseline evaluations included urinalysis, urine culture, and coagulation tests. All patients received a single intravenous dose of cefazolin sodium (1 g) and rectal cleansing with a fleet enema before biopsy, in accordance with our institutional protocol. No routine oral antibiotics were prescribed post-biopsy. For the procedure, an extended lithotomy posture was adopted, with the scrotum gently retracted using adhesive tape to facilitate exposure of the perineum, which was subsequently prepared with an iodine-based antiseptic. Transrectal ultrasonographic guidance was achieved using a biplanar probe capable of both sagittal and transverse imaging. The probe included a transperineal needle guide (models GTK154/GTK155, Geotek, Ankara, Turkey) enabling height and angle adjustment. The perineal skin entry site was selected according to prostate size and anatomy, typically located approximately 15 mm superior to the anal verge and 15 mm lateral to the midline. Local anesthesia was administered in two steps. First, local anesthesia was initiated by subcutaneous infiltration of 2 mL of 1% lidocaine using a 22-gauge needle. Subsequently, a 15-gauge, 10 cm coaxial Chiba needle (Geotek, Ankara, Turkey) was positioned adjacent to the skin surface without penetrating it. Through this guide, a 22-gauge, 20 cm Chiba needle was used to infiltrate the path to the prostate apex with 10 mL lidocaine. An additional 5 mL of anesthetic was applied directly at the apex. The same protocol was repeated contralaterally. Following anesthesia, the 15 G coaxial needle (Geotek, Ankara, Turkey) was inserted through the perineum and advanced within 10 mm of the prostate apex. This served as the access channel for all biopsies. Under dual-plane TRUS guidance, systematic 12-core biopsies were performed using an 18-gauge, 20 cm automated biopsy gun (Estacore Pro, Geotek, Ankara, Turkey). Additional 2–4 targeted cores were obtained from MRI-detected lesions. Sampling followed a fan-shaped approach, targeting both lateral and medial aspects of the apex, mid-gland, and base bilaterally. No sedation or intravenous analgesia was administered during the procedure. Oral paracetamol was prescribed for post-procedural pain management.

Biopsy samples were submitted in individually labeled containers per anatomical location. All specimens were evaluated by an experienced uropathologist. Pathology reports included Gleason score, tumor location, core length, number of cancer-positive cores, and percentage tumor involvement per core.

All procedures were conducted in the outpatient setting, and patients were discharged on the same day, provided they were able to void spontaneously. Patients were systematically assessed for the following complications: hematuria, acute urinary retention, urinary tract infection, fever, perineal pain, hematospermia, and perineal hematoma. No major complications were observed during the study period.

## 3. Statistical Analysis

Statistical analysis was performed using SPSS version 21.0 (IBM Corp., Armonk, NY, USA). Categorical variables were reported as frequencies and percentages. Numerical variables were summarized using median and quartiles (Q1–Q3). The Kolmogorov–Smirnov test was used to assess normality. Comparisons of dependent variables were performed using the Wilcoxon signed-rank test. A two-tailed *p*-value < 0.05 was considered statistically significant. Since the data did not follow a normal distribution according to the Kolmogorov–Smirnov test, numerical variables were presented as median and quartiles.

Longitudinal changes in questionnaire scores (IIEF, PEDT, and MSHQ-EJD) were evaluated using linear mixed-effects models to account for repeated measurements within individuals. Time, pathology group (benign vs. malign), and their interaction were included as fixed effects. Age, Charlson comorbidity index, and number of biopsy cores were entered as covariates. A random intercept for each participant was used to model within-subject correlation, and an autoregressive [AR(1)] covariance structure was applied. Models were estimated using restricted maximum likelihood (REML). Results are presented as regression coefficients with 95% confidence intervals, and a two-sided *p* value < 0.05 was considered statistically significant.

## 4. Results

The study cohort comprised 249 men who underwent transrectal ultrasound-guided TP prostate biopsy were included in the study. All included patients completed baseline and follow-up questionnaires at 1 and 3 months.

Baseline characteristics of the cohort are shown in Table 1. The median age was 66.0 (Q1–Q3 61.5–69.5) years, the median BMI was 25.8 (Q1–Q3 23.9–27.3), and the median Charlson Comorbidity Index was 2.0 (Q1–Q3 2.0–3.0). The median PSA value was 8.3 (Q1–Q3 5.6–12.2) ng/mL. The median number of cores was 12 (Q1–Q3 12.0–12.0). 14 cores were taken from 9 patients with lesions on MRI, 15 cores were taken from 1 patient, and 16 cores were taken from 3 patients. When patients were compared according to pathological diagnosis, those with prostate cancer (n = 132) tended to be slightly older and had higher Charlson Comorbidity Index scores compared with patients with benign pathology (n = 117), although these differences were not statistically significant (age, *p* = 0.296; Charlson Index, *p* = 0.337). Similarly, the prevalence of diabetes and hypertension was comparable between the two groups. (diabetes: 18.9% vs. 20.5%, *p* = 0.879; hypertension: 24.2% vs. 21.4%, *p* = 0.698).

### 4.1. Erectile Function—IIEF Questionnaire

Across the entire cohort, no significant change in total IIEF-5 scores was observed at month one (T2) and month three (T3) compared to baseline (T1): The median score remained 11.0 (Q1–Q3 8.0–13.0) at all time points (T1 vs. T2, *p* = 0.266; T1 vs. T3, *p* = 0.322) (Table 2).

### 4.2. Ejaculatory Function—PEDT and MSHQ-EjD Questionnaires

Across the entire patient group, PEDT and MSHQ-EjD scores did not change significantly over time. The median PEDT score was 11.0 (Q1–Q3 9.0–14.0) at T1 and remained similar at T2 and T3 (T1 vs. T2, *p* = 0.120; T1 vs. T3, *p* = 0.214). Similarly, MSHQ-EjD scores were stable at all time points (T1 vs. T2, *p* = 0.098; T1 vs. T3, *p* = 0.170) (Table 2).

In contrast, a statistically significant decrease in IIEF scores was found at the T2 and T3 time points in the subgroup of 132 patients diagnosed with prostate cancer (T1 vs. T2, *p* < 0.001; T1 vs. T3, *p* < 0.001). The median score in this group was measured as 11.0 (Q1–Q3 8.0–13.0) at T1, 10.0 (Q1–Q3 7.0–12.0) at T2, and 10.0 (Q1–Q3 8.0–12.0) at T3 (Table 3). A significant deterioration in ejaculatory function was also observed in this group. In this group, the PEDT score increased from 10.0 (Q1–Q3 8.0–13.0) at T1 to 12.0 (Q1–Q3 9.0–14.0/14.8) at T2 and T3 (*p* < 0.001). Likewise, the MSHQ-EjD score decreased from 13.0 (Q1–Q3 11.0–14.0) at T1 to 12.0 (Q1–Q3 10.0–13.0) at T2 and T3 (*p* < 0.001) (Table 3).

To further evaluate the independent effect of cancer diagnosis on sexual function outcomes while accounting for potential confounders, linear mixed-effects models were constructed for each outcome measure. The results of these analyses are presented in Table 4, Table 5 and Table 6.

Dependent variable: IIEF score

In the linear mixed-effects model, IIEF scores significantly decreased over time (β = −0.56, 95% CI −0.70 to −0.42, *p* < 0.001). While pathology status alone was not independently associated with IIEF score (*p* = 0.223), a significant time × pathology interaction was observed (β = 1.00, 95% CI 0.79 to 1.21, *p* < 0.001), indicating different longitudinal trajectories between benign and malign pathology groups (Table 4).

Increasing age was independently associated with lower IIEF scores (β = −0.25 per year, *p* < 0.001), whereas Charlson comorbidity index and number of biopsy cores were not significantly associated with IIEF outcomes.

Dependent variable: PEDT score

In the linear mixed-effects model, PEDT scores significantly increased over time (β = 0.88, 95% CI 0.75 to 1.00, *p* < 0.001). Pathology status was independently associated with higher PEDT scores, with patients in the benign group exhibiting higher scores compared to the malign group (β = 3.54, 95% CI 2.56 to 4.52, *p* < 0.001). A significant time × pathology interaction was observed (β = −2.02, 95% CI −2.20 to −1.84, *p* < 0.001), indicating divergent longitudinal changes in PEDT scores between pathology groups (Table 5).

Age, Charlson comorbidity index, and number of biopsy cores were not independently associated with PEDT scores.

Dependent variable: MSHQ-EJD score

In the linear mixed-effects model, MSHQ-EJD scores showed a significant decline over time (β = −0.52, 95% CI −0.62 to −0.42, *p* < 0.001). Pathology status was independently associated with MSHQ-EJD scores, with patients in the benign pathology group exhibiting lower scores compared with those in the malign group (β = −1.97, 95% CI −2.67 to −1.27, *p* < 0.001). A significant time × pathology interaction was observed (β = 0.97, 95% CI 0.82 to 1.11, *p* < 0.001), indicating different longitudinal trajectories of ejaculatory function between pathology groups (Table 6).

Higher Charlson comorbidity index was independently associated with lower MSHQ-EJD scores (β = −0.57, 95% CI −0.94 to −0.20, *p* = 0.003), whereas age and number of biopsy cores were not significantly associated with the outcome.

## 5. Discussion

Erectile dysfunction is a frequently reported and significant problem in the lifetime of prostate cancer sufferers. Psychological stress, anxiety, interruptions, and disruptions, which begin after diagnosis, can trigger sexual dysfunction [15]. This prospective study, which is thought to be the first to evaluate the effect of transperineal prostate biopsy on sexual function, showed that TP prostate biopsy had no significant effect on ejaculation and erectile function. However, patients diagnosed with prostate cancer demonstrated significant deterioration in sexual function.

The demographic characteristics of our cohort are noteworthy: the median age was 66 years and the median Charlson Comorbidity Index was 2.0. Advanced age and the presence of comorbidities such as diabetes, hypertension, and obesity are well-established risk factors for erectile dysfunction, even before any intervention [3]. In a cohort of 17,250 men evaluated before treatment, Pellegrino et al. found that the prevalence of ED in healthy men increased from approximately 20% to 68% between the ages of 50 and 75, but in men with comorbidities, these rates rose to 41% and 85%, respectively [16].

The stability of sexual function scores over time across the entire cohort suggests that transperineal biopsy does not directly impact sexual function. This finding is consistent with existing literature reporting lower complication rates and a reduced risk of neurovascular injury with transperineal biopsy compared to the transrectal approach [17]. A systematic review and meta-analysis found that erectile function is generally only temporarily impaired after prostate biopsy, with recovery typically occurring within three months [18]. In 2022, prospective cohort study by Napolitano et al., patients who underwent transrectal ultrasound-guided biopsy demonstrated a statistically significant decrease in IIEF-15 scores at one month post-procedure [6]. Although partial recovery was observed at the three-month follow-up, erectile and ejaculatory functions did not fully return to baseline levels. These findings suggest that the transrectal approach may have measurable and potentially prolonged adverse effects on male sexual function, particularly in the short- to mid-term period [6]. In another study, it was found that transrectal biopsy did not significantly affect the IIEF score or ED severity, most of the patients with decreased scores were in the group diagnosed with prostate cancer, and when grouped according to pathology results, the change in the IIEF score was statistically significant in the group diagnosed with prostate cancer [19].

In a recent prospective study evaluating the impact of ejaculation prior to transrectal prostate biopsy, patients were categorized into two groups: those who engaged in sexual activity with ejaculation before the procedure and those who abstained. No significant difference in erectile function was observed between these groups; however, a higher incidence of hematospermia and hematochezia was reported among sexually active individuals [19]. In contrast, our transperineal approach yielded minimal short-term complications and demonstrated stable sexual function outcomes in the overall cohort. These findings further support the favorable safety profile of transperineal biopsy, particularly when considering patient-reported outcomes.

The relationship between prostate cancer and erectile function has been extensively described in previous studies. A systematic review and meta-analysis published in 2020 by Fainberg et al. reported a mean decrease of 2.2 points in IIEF-5 scores at 1 month but noted that this change disappeared by months 3 and 6. This finding is consistent with the early, transient decrease observed in our study. Specifically, baseline EF scores at 3 months were demonstrated, supporting a significant reversal of this dysfunction [17]. Similarly, a recent meta-analysis by Ermis et al. comparing transrectal and transperineal approaches demonstrated that erectile dysfunction following prostate biopsy is transient, with function typically returning to baseline by 6 months regardless of the technique used [20]. While the study by Morelli et al. reported continued deterioration in some IIEF subdomains at 3 months after TRUS-Bx, this deterioration was observed only in PCa patients in our study using TP-Bx [6]. Furthermore, while some studies have reported long-term sexual dysfunction after TRUS-Bx, our TP-Bx cohort demonstrated stable sexual function at 3 months. However, as this is a single-arm study, direct comparison with the transrectal approach is not possible, and randomized comparative trials are needed to confirm any potential differences between the two techniques. No significant change in sexual function was detected in the other studies after transperineal biopsy [6,21]. This is consistent with the findings of Fainberg et al. This supports the notion that post-biopsy erectile dysfunction is transient and generally minimal, as reported in a meta-analysis [17]. However, our study stands out in the literature for demonstrating a significant deterioration in sexual function scores in patients diagnosed with prostate cancer. Although the decline in sexual function scores among prostate cancer patients reached statistical significance, the absolute change was modest and may not necessarily represent a clinically meaningful deterioration. The minimal clinically important difference (MCID) for IIEF-5 has been estimated at approximately 4 points [22]. The observed 1-point median decrease in our cancer subgroup (from 11 to 10) falls below this threshold, suggesting that while the change is statistically detectable, it may not represent a clinically perceptible deterioration from the patient’s perspective. However, MCID values have not been specifically established in the post-biopsy setting, and individual patient experiences may vary. This finding suggests that post-diagnosis psychological stress and disease awareness may affect sexual function.

To further evaluate whether the observed decline in sexual function among cancer patients was independent of potential confounders, multivariable linear mixed-effects models were performed for each outcome. After adjusting for age, Charlson comorbidity index, and number of biopsy cores, a significant time × pathology interaction was observed for IIEF-5, PEDT, and MSHQ-EjD. These results indicate that patients with prostate cancer exhibited significantly different longitudinal trajectories compared with those with benign pathology, independent of demographic and procedural factors. This supports the notion that cancer diagnosis itself, likely mediated through psychological mechanisms, is the primary driver of sexual function deterioration in this population.

These findings are qualitative and suggest that libido and physical performance are impaired after cancer diagnosis, independent of behavioral factors [23,24]. The literature also supports the hypothesis that psychological stress and sexual self-perception are important factors contributing to post-biopsy sexual dysfunction [25]. Although we did not formally assess anxiety or depression, the significant decline in sexual function observed in patients diagnosed with cancer in studies using validated pre-biopsy anxiety measures is likely influenced by emotional stress [7,26]. While the use of validated anxiety scoring systems provides insight into procedural stress, our observations of post-diagnostic sexual dysfunction in cancer-positive patients further emphasize the need for integrated psychosocial support protocols.

The strengths of this study include its prospective design, multiple time-point assessments using standardized questionnaires, and the inclusion of an adequate sample size. However, several limitations should be acknowledged. First, follow-up was limited to three months, which may be insufficient to capture long-term recovery patterns. Secondly, validated psychological assessment instruments were not administered at baseline. Consequently, we cannot definitively attribute the observed decline in sexual function among cancer patients to psychological distress versus physiological mechanisms related to the biopsy procedure itself; this association should therefore be considered hypothesis-generating rather than confirmatory. Of note, psychological assessment has been incorporated into our ongoing follow-up protocol, and these data will be reported in a subsequent study examining medium- to long-term outcomes. Third, although multivariable mixed-effects models were used to adjust for age, comorbidity burden, and procedural factors, residual confounding from unmeasured variables (e.g., baseline anxiety, relationship quality, or partner factors) cannot be entirely excluded. However, confounding effects of prostate cancer treatment were minimized by excluding all patients who received active therapy during the three-month observation period. Fourth, patients using PDE5 inhibitors were excluded to ensure a homogeneous cohort; however, this may limit the generalizability of our findings to the broader population of men undergoing prostate biopsy, many of whom use these medications.

## 6. Conclusions

TP biopsy was not associated with significant short-term changes in sexual function scores in this cohort. Men diagnosed with prostate cancer showed statistically significant score declines; however, this may represent a transient decline, and long-term follow-up is necessary to determine the persistence of these changes.

## Figures and Tables

**Table 1:** Patient characteristics.

Characteristic	(n = 249)
Age (years), Median (Q1–Q3)	66.0 (61.5–69.5)
Body Mass Index, Median (Q1–Q3)	25.8 (23.9–27.3)
Charlson score, Median (Q1–Q3)	2.0 (2.0–3.0)
Diabetes, n (%)	49 (19.7)
Hypertension, n (%)	57 (22.9)
Prostate cancer, n (%)	132 (53.0)
Anticoagulant therapy, n (%)	29 (11.6)
Antiaggregant therapy, n (%)	18 (7.2)
Alpha-1 adrenergic receptor blockers therapy, n (%)	6 (2.4)
5-alpha reductase inhibitors therapy, n (%)	25 (10.0)
PSA, Median (Q1–Q3)	8.3 (5.6–12.2)
Biopsy cores, Median (Q1–Q3)	12.0 (12.0–12.0) range: 12–16

Q1: First quartile; Q3: Third quartile; PSA: Prostate specific antigen.

**Table 2:** International Index of Erectile Function (IIEF) Questionnaire and Ejaculatory function questionnaires administrated to 249 patients before prostate biopsy (T1), 1 month after (T2) and 3 months after (T3).

					Wilcoxon Signed Ranks Test *p*-Value	
		T1	T2	T3	T1 vs. T2	T1 vs. T3
IIEF	Median (Q1–Q3)	11.0 (8.0–13.0)	11.0 (8.0–13.0)	11.0 (8.0–13.0)	0.266	0.322
PEDT	Median (Q1–Q3)	11.0 (9.0–14.0)	11.0 (8.0–14.0)	11.0 (8.0–14.0)	0.120	0.214
MSHQ-EjD	Median (Q1–Q3)	12.0 (10.0–14.0)	12.0 (10.0–14.0)	12.0 (10.0–14.0)	0.098	0.170

PEDT: Premature Ejaculation Diagnostic Tool; MSHQ-EjD: Male Sexual Health Ejaculatory Dysfunction Questionnaire-Ejaculatory Dysfunction; Q1: First quartile; Q3: Third quartile.

**Table 3:** Changes in IIEF, PEDT, and MSHQ-EjD Scores in Patients Diagnosed With Prostate Cancer before prostate biopsy (T1), 1 month after (T2) and 3 months after (T3).

					Wilcoxon Signed Ranks Test *p*-Value	
		T1	T2	T3	T1 vs. T2	T1 vs. T3
IIEF	Median (Q1–Q3)	11.0 (8.0–13.0)	10.0 (7.0–12.0)	10.0 (8.0–12.0)	<0.001	<0.001
PEDT	Median (Q1–Q3)	10.0 (8.0–13.0)	12.0 (9.0–14.0)	12.0 (9.0–14.8)	<0.001	<0.001
MSHQ-EjD	Median (Q1–Q3)	13.0 (11.0–14.0)	12.0 (10.0–13.0)	12.0 (10.0–13.0)	<0.001	<0.001

PEDT: Premature Ejaculation Diagnostic Tool; MSHQ-EjD: Male Sexual Health Ejaculatory Dysfunction Questionnaire-Ejaculatory Dysfunction; Q1: First quartile; Q3: Third quartile.

**Table 4:** Linear mixed-effects model for IIEF score.

Variable	β	95% CI	*p* Value
Time	−0.56	−0.70–−0.42	<0.001
Pathology (benign vs. malignant)	−0.55	−1.43–0.34	0.223
Time × Pathology	1.00	0.79–1.21	<0.001
Age (years)	−0.25	−0.33–−0.17	<0.001
Charlson comorbidity index	−0.18	−0.63–0.27	0.435
Number of biopsy cores	−0.19	−0.85–0.46	0.559

Model: Linear mixed-effects model with random intercept for subject and AR(1) covariance structure.

**Table 5:** Linear mixed-effects model for PEDT score.

Variable	β	95% CI	*p* Value
Time	0.88	0.75–1.00	<0.001
Pathology (benign vs. malignant)	3.54	2.56–4.52	<0.001
Time × Pathology	−2.02	−2.20–−1.84	<0.001
Age (years)	−0.06	−0.15–0.03	0.211
Charlson comorbidity index	−0.20	−0.73–0.33	0.455
Number of biopsy cores	0.59	−0.17–1.36	0.129

Model: Linear mixed-effects model with random intercept for subject and AR(1) covariance structure.

**Table 6:** Linear mixed-effects model for MSHQ-EJD score.

Variable	β	95% CI	*p* Value
Time	−0.52	−0.62–−0.42	<0.001
Pathology (benign vs. malignant)	−1.97	−2.67–−1.27	<0.001
Time × Pathology	0.97	0.82–1.11	<0.001
Age	0.02	−0.05–0.08	0.584
Charlson comorbidity index	−0.57	−0.94–−0.20	0.003
Number of biopsy cores	−0.01	−0.54–0.53	0.978

Model: Linear mixed-effects model with random intercept for subject and AR(1) covariance structure.

## Data Availability

The data presented in this study are available on request from the corresponding author due to ethical reasons.
